# Cross-Sectional Study of Rehabilitation Training Intensity and Physical Restraints in Patients With Cardiovascular Disease and Neurocognitive Disorders in Acute Care Hospitals

**DOI:** 10.7759/cureus.102937

**Published:** 2026-02-04

**Authors:** Yukako Ishida, Tetsuro Kitamura, Masatsugu Ikeuchi, Megumi Matsuda, Kazuhiko Yamamuro, Yasuyo Kobayashi, Yusuke Inagaki, Shungo Hikoso, Mitsuharu Hosono, Takashi Okada, Akira Kido

**Affiliations:** 1 Department of Community-Based Rehabilitation Systems Science, Nara Medical University, Kashihara, JPN; 2 Department of Rehabilitation Medicine, Nara Medical University, Kashihara, JPN; 3 Department of Neurosugery, Nara Medical University Hospital, Kashihara, JPN; 4 Department of Nursing, Psychiatric Center, Nara Medical University Hospital, Kashihara, JPN; 5 Center for Health Control, Nara Medical University Hospital, Kashihara, JPN; 6 Department of Psychiatry, Nara Medical University, Kashihara, JPN; 7 Department of Cardiovascular Medicine, Nara Medical University, Kashihara, JPN; 8 Department of Thoracic and Cardiovascular Surgery, Nara Medical University, Kashihara, JPN

**Keywords:** dementia care team, neurocognitive disorders, patients with cardiovascular disease, physical restraints, rehabilitation

## Abstract

Background

Previous studies have reported the characteristics of rehabilitation therapy and physical restraint use in patients with neurocognitive disorders during the acute phase, based on three years of clinical records from dementia care team rounds at our hospital. The incidence of neurocognitive disorders varies by disease group, and the degree of physical restraint is significantly associated with the intensity of the rehabilitation therapy. Among the disease groups addressed in this study, we were particularly interested in cardiovascular diseases. In patients with cardiovascular disease, which is associated with high clinical acuity and disease severity, the presence of neurocognitive disorders may affect the prognosis. This study aimed to clarify the incidence of neurocognitive impairment (including delirium) in patients with cardiovascular disease and its characteristics with respect to rehabilitation therapy and physical restraint.

Methods

We examined data from dementia care team rounds conducted between July 1, 2021, and October 7, 2024, to identify relationships between clinicopathological characteristics, rehabilitation therapy, and physical restraint status. Cardiovascular diseases were classified into nine subgroups: ischemic diseases; arrhythmia; heart failure; valvular heart disease; acute pericarditis; aortic diseases; peripheral vascular diseases; and renal, carotid, and subclavian artery diseases. The treatment modalities were categorized as conservative management, pacemaker implantation (PM), percutaneous coronary intervention (PCI), minimally invasive surgery (MIS; including endovascular aneurysm repair and endovascular thoracic aortic repair), thoracotomy, and laparotomy. Cognitive function was assessed weekly, and patients whose neurocognitive disorders improved sufficiently were excluded from the dementia care team. Chi-square (χ²) tests were used to assess the associations between the variables.

Results

A total of 101 patients with cardiovascular disease underwent 379 rounds. Significant differences in treatment selection were observed between the disease groups (p < 0.0001). Significant differences in the number of dementia rounds were also observed between treatment groups, with more rounds occurring in patients who received invasive treatment (p = 0.019). No significant differences in the number of neurocognitive disorder episodes, training intensity, or physical restraint intensity were identified between the treatment groups. Patients who received stronger physical restraints generally underwent high-intensity training (p = 0.015).

Conclusions

Patients with cardiovascular diseases who received more invasive treatments generally received more dementia care team rounds, suggesting a possible association with prolonged neurocognitive disorders. Most patients who were subjected to stronger physical restraints could undergo intensive rehabilitation, suggesting that increased staffing levels could reduce the need for physical restraints.

## Introduction

Japan, now facing the era of a super-aging society, has experienced an increase in the number of older patients with dementia, even in acute care hospitals [1,2]. However, acute care hospitals are generally not designed with dementia care as their primary purpose [3], and patients are typically not admitted specifically for dementia treatment [4], indicating that dementia is not a priority for care or treatment [3,4]. To provide appropriate medical care to older patients with dementia in acute care hospitals, the 2016 revision of Japan’s health insurance system introduced a new dementia care add-on fee as an incentive within the basic hospitalization fee [5-7]. Furthermore, the 2024 medical fee revision emphasizes the need to minimize physical restraints and improve delirium management in dementia care [8]. Patients with dementia are at a high risk of developing delirium, and the clinical distinction between the two is often difficult [9]. Therefore, in acute care settings, conceptualizing and managing this condition as a neurocognitive disorder that encompasses both dementia and delirium is practical [10].

Our hospital initiated the dementia care team rounds in April 2017. As a unique initiative, rehabilitation physicians, physical therapists, and occupational therapists joined the dementia care team in April 2021. This was based on our policy of using the active or passive control of a patient’s physical activity, including both physical restraint and training, as key indicators of acute-phase management during these rounds [10]. Based on this policy, data collection on physical impairment and rehabilitation therapy for the patients included in the rounds commenced, revealing new aspects of dementia care [10].

Disease-specific characteristics may influence clinical management during the acute phase in patients with neurocognitive disorders. In our previous study based on dementia care team rounds, the incidence of neurocognitive disorders varied across disease groups defined by rehabilitation classifications, and the degree of physical restraint was significantly associated with rehabilitation training intensity [10,11]. Among these, cardiovascular disease is notable because it represents a relatively homogeneous group with high acuity and severity. Although open-heart surgery is a well-established risk factor for delirium [12,13], neurocognitive disorders may also occur during the treatment of major cardiovascular diseases, potentially affecting clinical outcomes and prognosis.

The primary aim of this study was to clarify the incidence and characteristics of neurocognitive disorders in patients with cardiovascular disease in acute care hospitals. The secondary aim was to examine the association between these disorders and rehabilitation training and physical restraint intensities. We conducted a subgroup analysis of the data from previously reported dementia care team rounds.

## Materials and methods

Study setting and dementia care team rounds

This study was conducted at a single acute care hospital in Japan. Dementia care team rounds were implemented in April 2017 in accordance with the 2016 revision of Japan’s health insurance system, which introduced a dementia care add-on fee to promote appropriate dementia care in acute care hospitals [5-7]. This system requires a multidisciplinary team comprising dedicated physicians and nurses with specialized training and experience in dementia care, as well as social or mental health welfare workers involved in discharge coordination. The team is also required to engage in continuous quality improvement activities, including weekly care conferences, ward rounds, and consultations with ward staff [5,6].

Patients were identified through weekly evaluations of all inpatients, which were conducted by the assigned ward nurses, overseen by a certified dementia care nurse, and confirmed by a psychiatrist within the team. Since April 2021, rehabilitation physicians, physical therapists, and occupational therapists have joined the dementia care team to comprehensively assess physical activity, rehabilitation therapy, and physical restraint use. Data on physical function, rehabilitation therapy, and physical restraint were systematically collected from the patients included in the rounds.

Patients included in the study

This study utilized data from 1,987 rounds (580 patients) conducted by the dementia care team between July 1, 2021, and October 7, 2024. Information regarding age, sex, diagnosis, treatment, rehabilitation content, and physical restraint status was extracted from the medical records and analyzed. Written informed consent was obtained from all the patients. This study was approved by the Ethics Committee of Nara Medical University (approval no. 3913).

**Figure 1:**
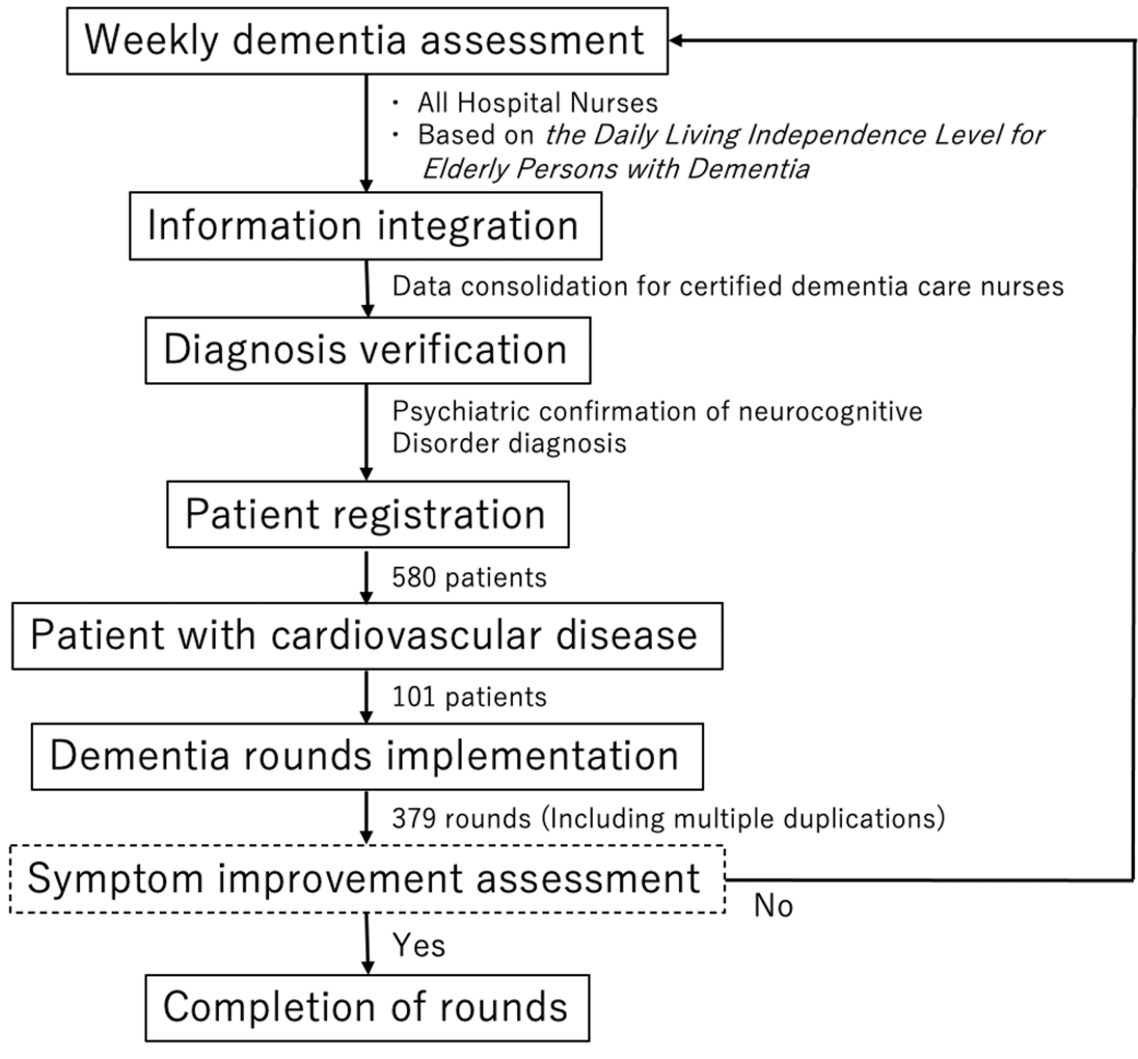
Flowchart of the case enrollment process Daily Living Independence Level for Elderly Persons with Dementia [14]

Patients eligible for dementia care team rounds were selected based on the diagnosis of a certified dementia care nurse, according to the Daily Living Independence Level for Elderly Persons with Dementia [14]. Cognitive function assessments were conducted weekly for all hospitalized patients aged ≥65 years. These assessments were overseen by a certified dementia care nurse, performed by each patient’s assigned nurse, and verified by a psychiatrist within the team (Figure 1). The inclusion criterion for the rounds was Grade III or higher neurocognitive disorders that impeded acute treatment. Adult patients aged <65 years were also included if their impairment was judged to impede acute treatment based on this criterion. Meanwhile, patients aged <20 years, those who had experienced a life-threatening event, and those scheduled for discharge before the next round were excluded from the study. Diagnosis by a certified dementia care nurse was confirmed by a psychiatrist, according to the Diagnostic and Statistical Manual of Mental Disorders, Fifth Edition (American Psychiatric Association) [15]. Only patients who were flagged by a dementia care nurse for possible neurocognitive disorders were subsequently reviewed and verified by a psychiatrist within the team. Screening was performed weekly. If a patient’s neurocognitive disorder was judged to have improved to a degree that no longer impeded the management of the underlying condition, the patient was removed from the rounds. Conversely, if no improvement in impairment was observed, the rounds continued. All patients with neurocognitive disorders, including dementia and delirium, were included in the analysis.

Classification of disease groups

The disease-specific rehabilitation classification categorizes all diseases into six groups: musculoskeletal diseases, cancer, respiratory diseases, cardiovascular diseases, cerebrovascular diseases, and disuse syndrome [11]. A subgroup analysis was performed for patients with cardiovascular diseases. Cardiovascular diseases were classified into nine subgroups: 1) ischemic diseases; 2) arrhythmia; 3) heart failure; 4) valvular heart disease; 5) acute pericarditis; 6) aortic diseases; 7) peripheral vascular diseases; 8) renal artery, carotid artery, and subclavian artery diseases (classified as cardiovascular diseases in the disease-specific rehabilitation classification); and 9) others. The treatment modalities were categorized as follows: conservative management, pacemaker implantation (PM), percutaneous coronary intervention (PCI), minimally invasive surgery (MIS) (including endovascular aneurysm repair and endovascular thoracic aortic repair), thoracotomy, and laparotomy. All MIS procedures were analyzed together as a single category.

Rehabilitation treatment

In this study, “rehabilitation therapy” refers to structured interventions provided by rehabilitation therapists, whereas “physical activity” refers to the overall physical movement of patients during daily activities. Information on rehabilitation treatment was extracted from medical records for ambulation training, muscle-strengthening training, feeding and swallowing function training, and cognitive function training. Ambulation training was analyzed in five stages: 1) therapeutic (in-bed) exercise, 2) bed mobility (supine-to-sit) training, 3) transfer training, 4) standing training, and 5) gait training. Stage 0 indicated that the patient did not receive rehabilitation intervention. Patients were classified into only one ambulation training stage per assessment. In this study, the ambulation stage was used as an indicator of rehabilitation training intensity, reflecting the highest level of mobility training performed during each assessment, rather than the overall duration or frequency of rehabilitation therapy.

Physical restraints

We reviewed five types of restraints from medical records: 1) floor sensor mats, 2) pull-string alarms, 3) bedside rails, 4) hand-restraint mittens, and 5) trunk restraints (belts). We classified types 1 and 2 as mild restraints (I) and types 3, 4, and 5 as severe restraints (II) based on their impact on patients’ physical movement. Mild restraints minimally restrict movement, whereas severe restraints substantially restrict mobility. Stage 0 indicates the absence of physical restraint.

Statistical analysis

Statistical analyses were performed using the JMP Pro version 17.2.0 (SAS Institute Inc., Cary, NC, USA). Chi-square (χ²) tests were performed for nominal or ordinal variables for each disease group, ambulation training stage, and physical restraint category. No adjustments were made for repeated or multiple comparisons. Statistical significance was defined as p < 0.05.

## Results

Disease subgroups and treatments

**Figure 2:**
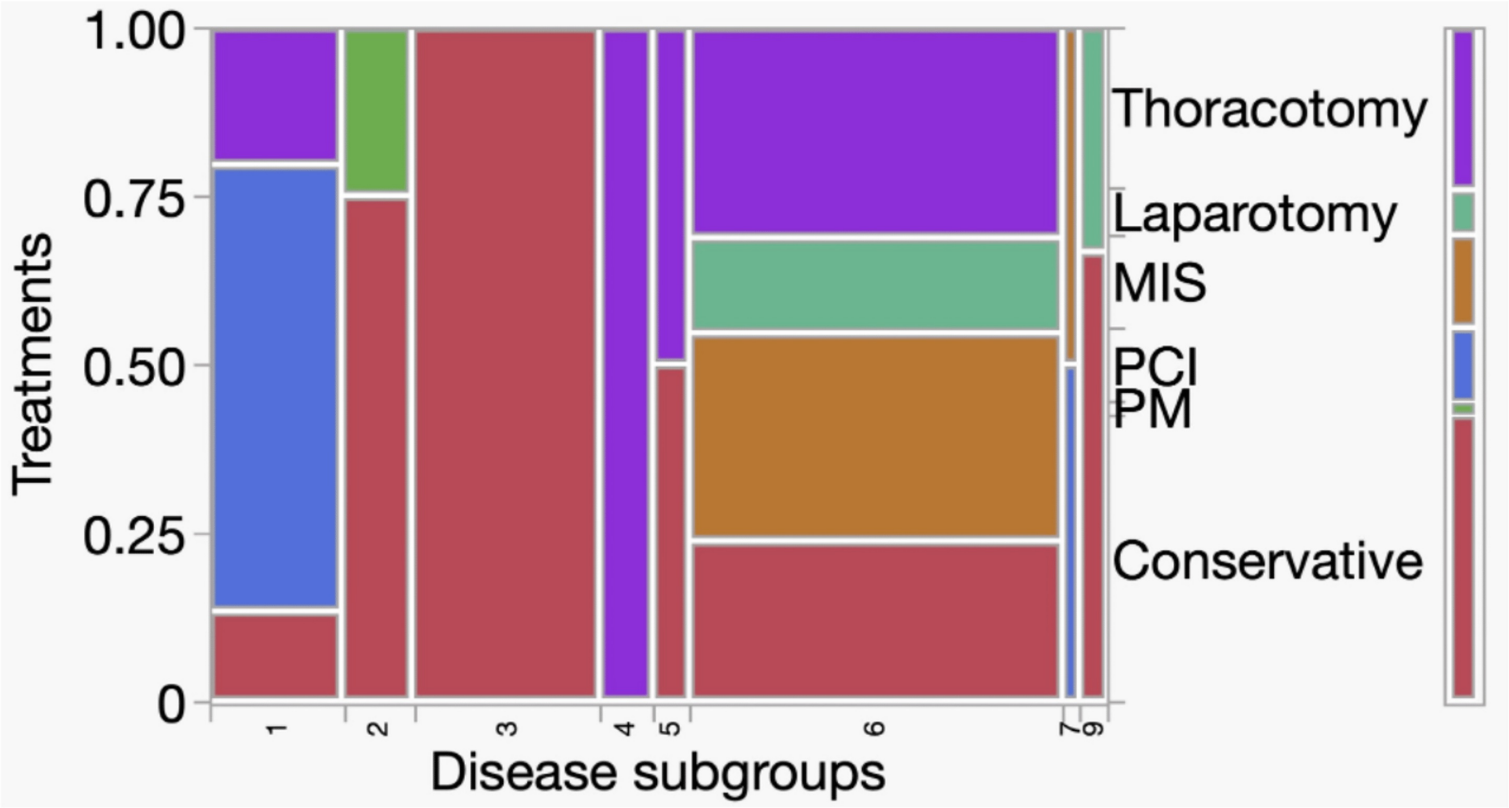
Mosaic plots for disease subgroups and treatment methods Abbreviations: PM, pacemaker implantation; PCI, percutaneous coronary intervention; MIS, minimally invasive surgery The left y-axis indicates the proportion of patients (range: 0–1.00). The treatment methods (conservative, PM, PCI, MIS, laparotomy, and thoracotomy) are shown as categorical labels. The x-axis represents the disease subgroups coded from 1 to 9: 1, ischemic diseases; 2, arrhythmia; 3, heart failure; 4, valvular heart disease; 5, acute pericarditis; 6, aortic diseases; 7, peripheral vascular diseases; 8, renal/carotid/subclavian artery diseases; and 9, others.

During the study period, 101 patients with cardiovascular and neurocognitive disorders met the study criteria and underwent 379 rounds of dementia care. Figure 2 presents a mosaic plot illustrating the distribution of treatment methods (conservative, PM, PCI, MIS, laparotomy, and thoracotomy) across disease subgroups 1-9. The left y-axis represents the proportion of patients, whereas the categorical labels indicate different treatment methods. The x-axis represents the disease subgroups coded from 1 to 9. The mosaic plot demonstrates how the treatment selection varied among the subgroups. Overall, conservative treatment was predominantly used in subgroup 3, PCI in subgroup 1, and thoracotomy in subgroups 4 and 5. A significant association was identified between treatment methods and disease subgroups (χ² = 160.38, df = 35, p < 0.001). No post hoc analyses were performed to identify pairwise differences between the subgroups. Specific patient counts and proportions for each treatment method within the disease subgroups can be visually interpreted from Figure 2. Treatment strategies should be tailored according to disease subgroups, reflecting the clinical characteristics and typical interventions for each category of cardiovascular disease.

Relationship between the onset of neurocognitive disorders and treatment groups

Considering that the severity of treatment invasiveness may be associated with the onset of neurocognitive disorders, we examined the ratio of patients who underwent dementia care team rounds to the total number of patients who received each treatment. A total of 1,608 patients underwent cardiovascular rehabilitation: 886 underwent conservative treatment, 57 PM, 106 PCI, 167 MIS, 77 laparotomy, and 315 thoracotomies. Table 1 shows the breakdown of the treatment groups, the total number of patients treated during this period, and their respective percentages. No statistically significant differences in the incidence of neurocognitive disorders were observed between the treatment groups.

**Table 1:** Number of patients receiving dementia care rounds and total number of patients in each treatment group

	Conservative	PM	PCI	MIS	Laparotomy	Thoracotomy
Dementia care rounds (%)	43 (4.9)	2 (3.5)	11 (10.4)	14 (8.4)	7 (9.1)	24 (7.6)
Total	886	57	106	167	77	315

Relationship between the number of dementia care rounds and treatment groups

**Figure 3:**
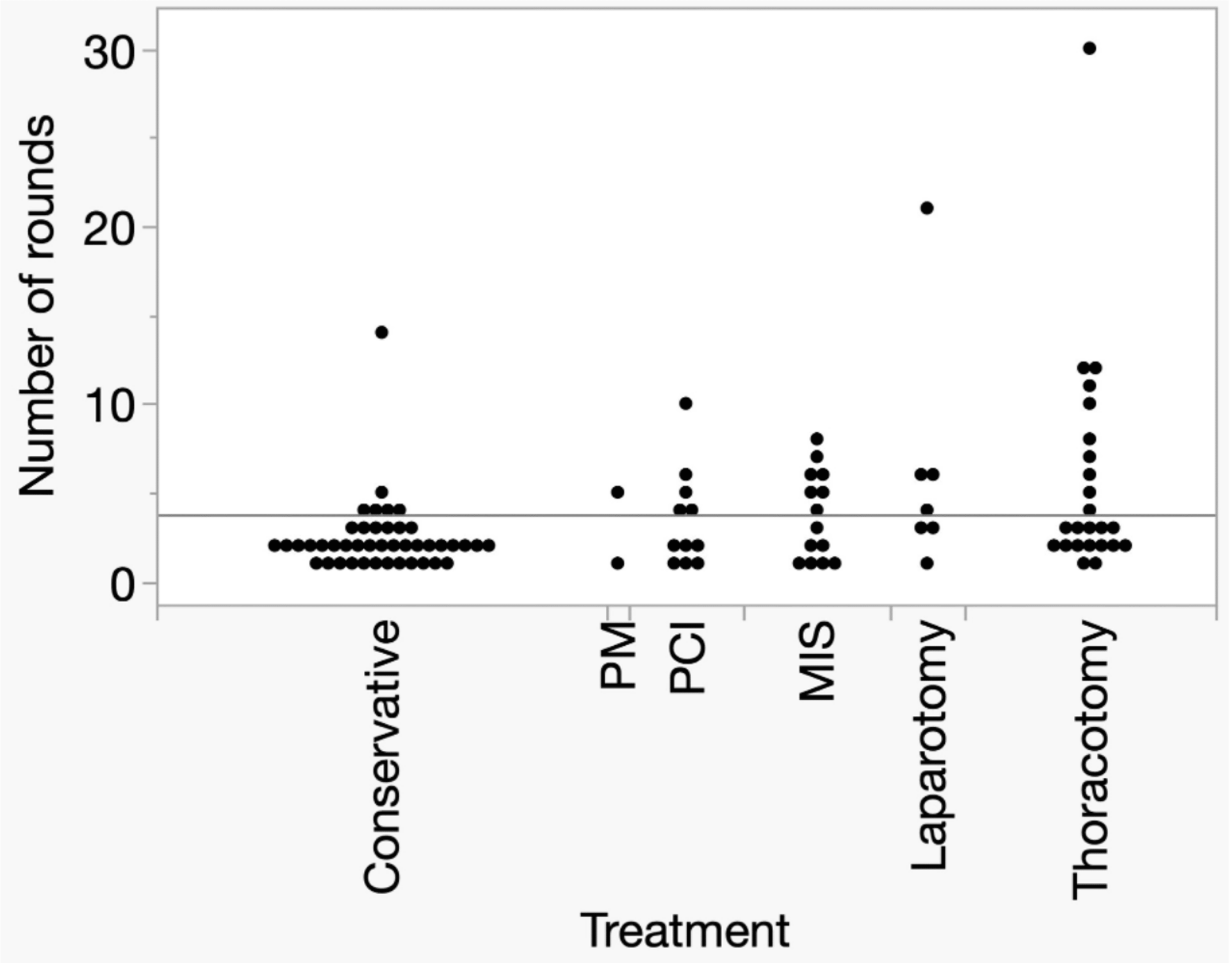
One-way analysis of variance for number of rounds by treatment Abbreviations: PM, pacemaker implantation; PCI, percutaneous coronary intervention; MIS, minimally invasive surgery

Figure 3 shows the number of dementia care team rounds for the patients in each treatment group. Patients who received more invasive treatments generally underwent more rounds, and statistically significant differences in the number of dementia rounds were observed among treatment groups (χ² = 13.55, df = 5, p = 0.019). Thus, patients undergoing more invasive treatments tended to undergo more rounds of dementia care.

Relationship between rehabilitation training intensity and treatment groups

**Figure 4:**
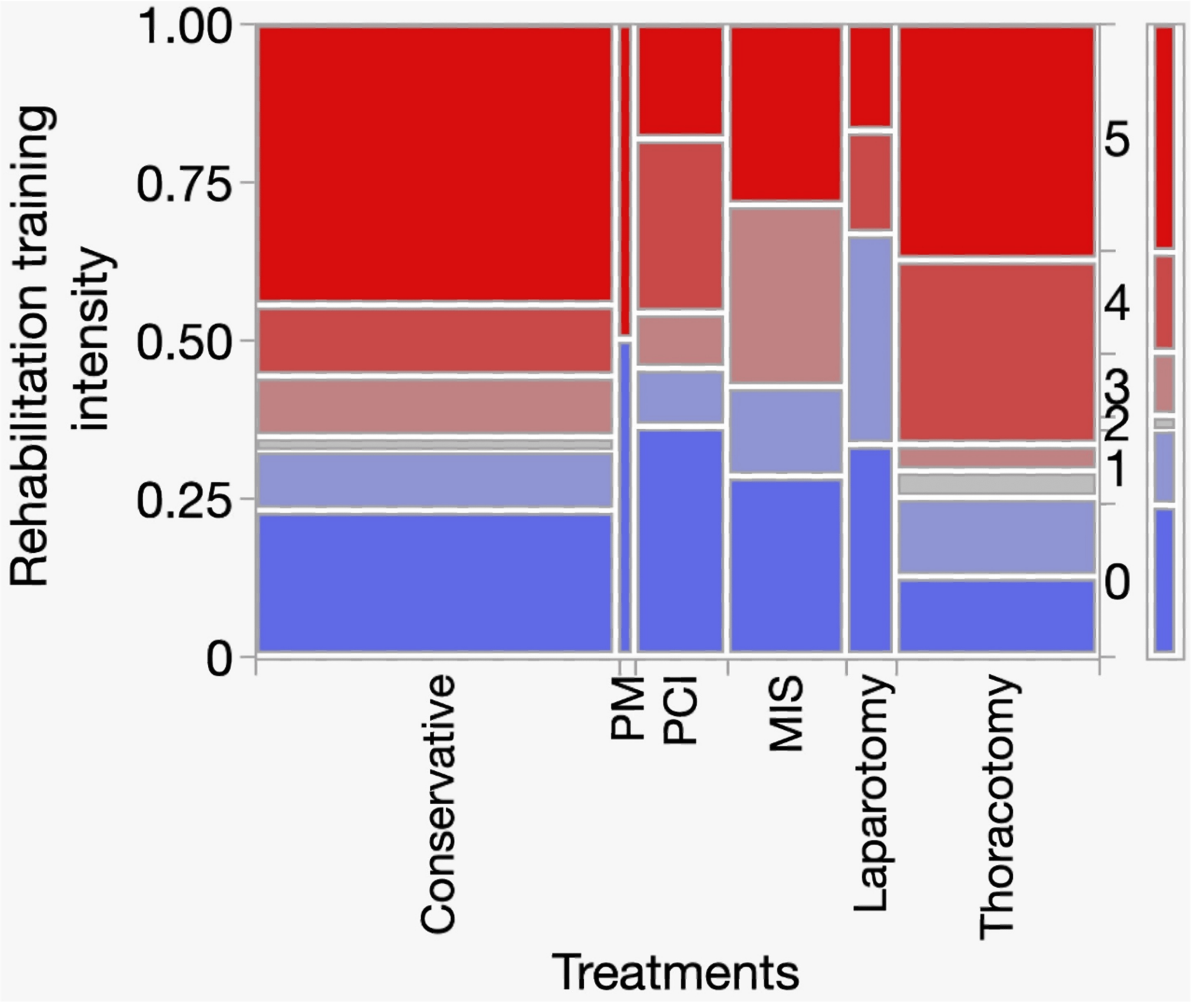
Analysis of the contingency table for treatment and rehabilitation training intensity Abbreviations: PM, pacemaker implantation; PCI, percutaneous coronary intervention; MIS, minimally invasive surgery The left y-axis represents the proportion of patients (0–1.00), and the x-axis shows the treatment methods. The column widths are proportional to the number of patients in each group. The right y-axis represents the rehabilitation training intensity stages (0–5): 0, none; 1, therapeutic exercise; 2, bed mobility training; 3, transfer training; 4, standing training; 5, gait training.

Figure 4 shows a mosaic plot of the rehabilitation training intensity in each treatment group. The left y-axis represents the proportion of patients (range, 0-1.00), and the x-axis represents the treatment methods. The width of each column indicates the number of patients in each treatment group. The level of rehabilitation intensity was coded as a categorical variable ranging from 0 to 5. Most patients in all treatment methods received stages 2-4 rehabilitation training. No significant differences in training intensity were observed between the treatment groups (χ² = 22.86, df = 25, p = 0.586). Thus, rehabilitation training intensity was generally similar regardless of the treatment method.

Relationship between physical restraint intensity and treatment groups

**Figure 5:**
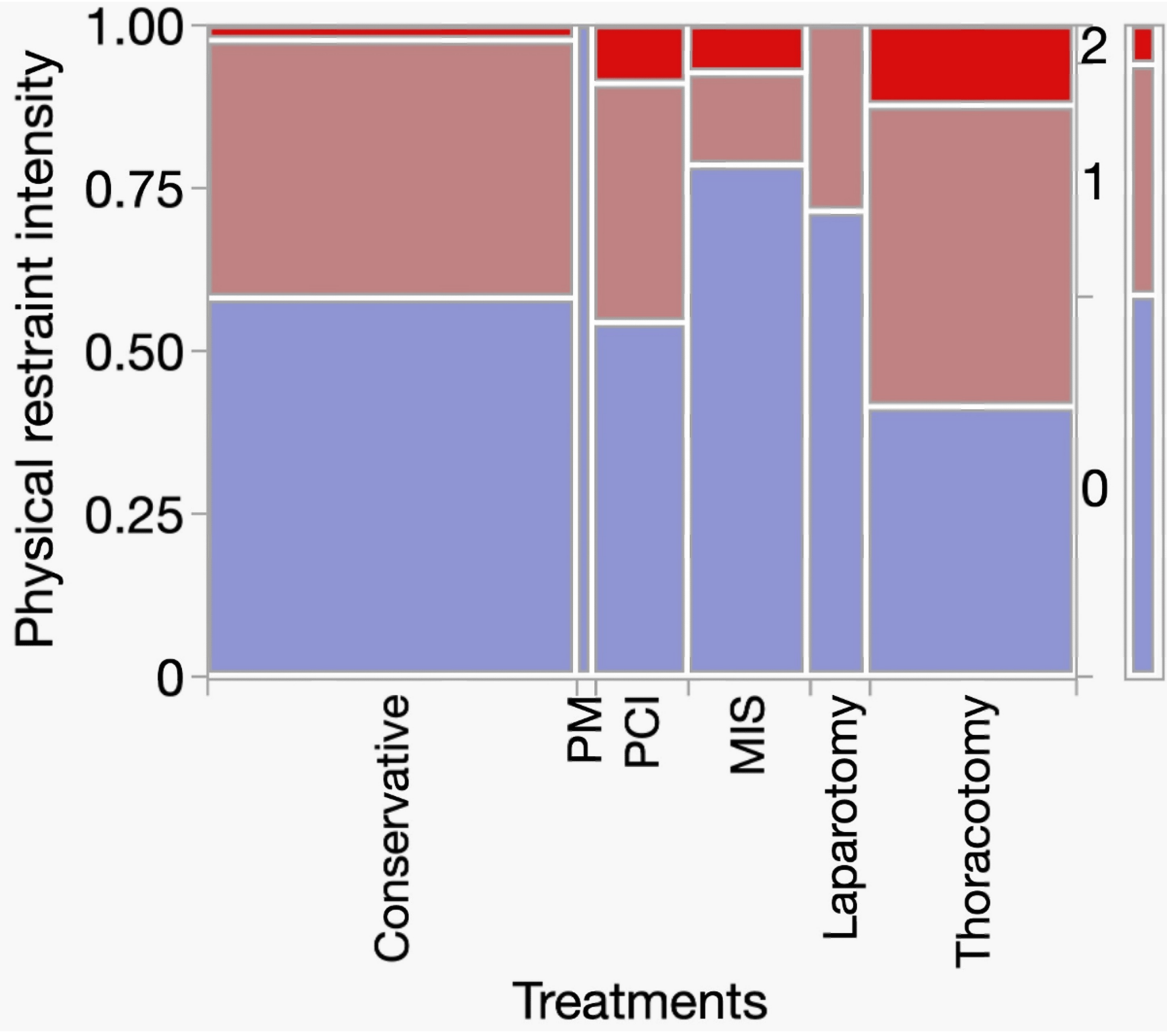
Analysis of contingency tables for treatment and physical restraint intensity Abbreviations: PM, pacemaker implantation; PCI, percutaneous coronary intervention; MIS, minimally invasive surgery The left y-axis shows the proportion of patients (0–1.00), and the right y-axis indicates the physical restraint levels, coded as 0 (none), 1 (mild restraint), and 2 (severe restraint). The x-axis represents the treatment method, and the column widths are proportional to the number of patients in each group.

Figure 5 shows a mosaic plot of the intensity of physical restraint in each treatment group. The left y-axis represents the proportion of patients subjected to physical restraint (range, 0-1.00), whereas physical restraint levels were coded as categorical variables from 0 to 2 (0 = none, 1 = mild restraint, and 2 = severe restraint), as indicated on the right side. The x-axis shows the treatment methods, and the width of each column corresponds to the number of patients in each treatment group. The mosaic plot demonstrates that most patients in all treatment groups were not restrained (level 0), whereas a small proportion experienced severe restraints (level 2). No significant differences in physical restraint intensity were observed among treatment groups (χ² = 9.88, df = 10, p = 0.452). The use and intensity of physical restraints were generally similar across treatment methods.

Relationship between rehabilitation training intensity and physical restraint intensity

**Figure 6:**
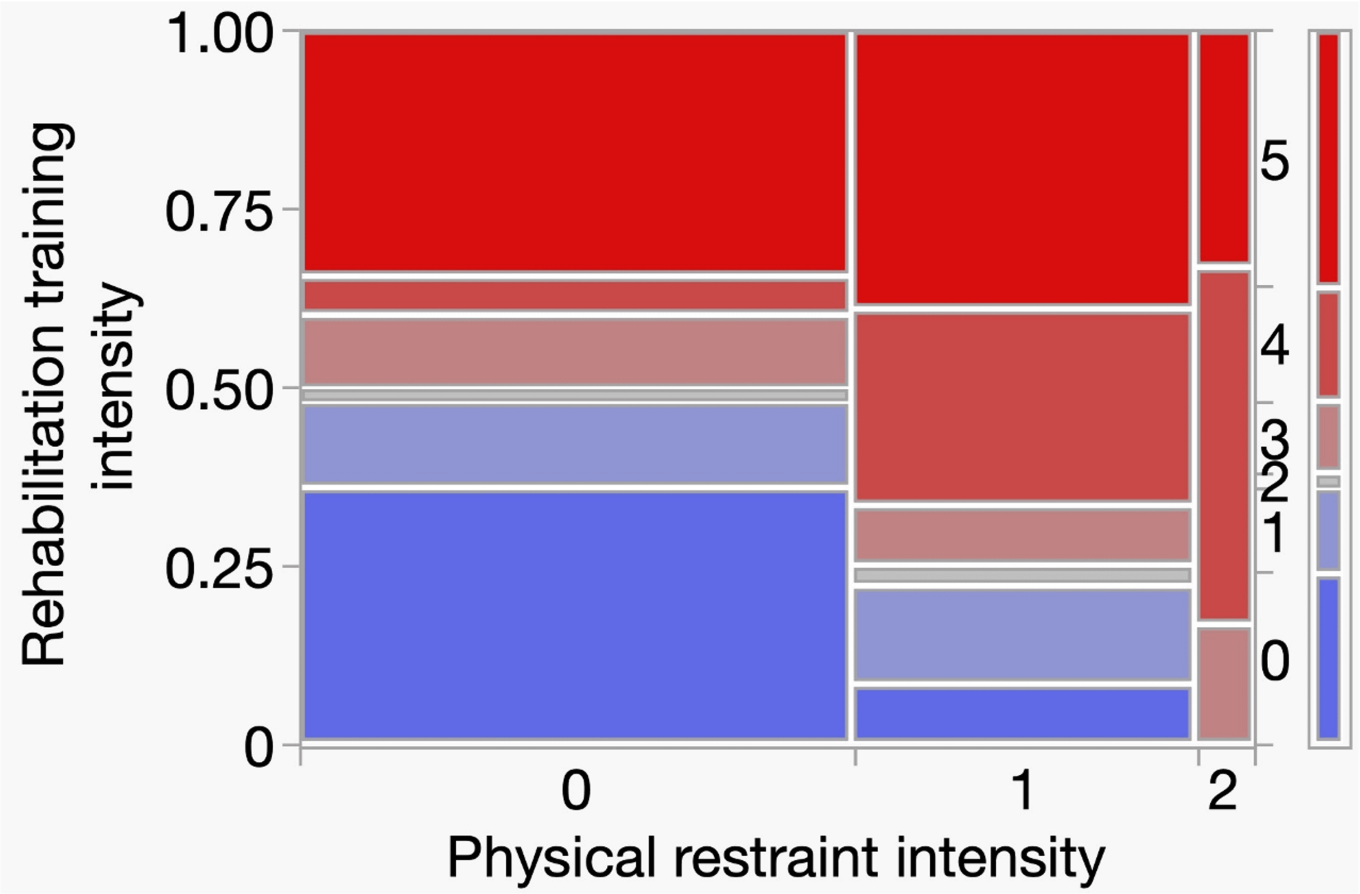
Analysis of contingency tables for restraint intensity and rehabilitation intensity The left y-axis indicates the proportion of patients according to rehabilitation intensity (0–1.00). The right y-axis represents the rehabilitation training intensity stages (0–5): 0, none; 1, therapeutic exercise; 2, bed mobility training; 3, transfer training; 4, standing training; and 5, gait training. The x-axis represents physical restraint levels (0–2): 0, none; 1, mild; and 2, severe.

Figure 6 shows a mosaic plot of the relationship between rehabilitation training intensity and physical restraint intensity. The left y-axis represents the proportion of patients, the right side indicates the rehabilitation intensity levels (0-5), and the x-axis represents the physical restraint categories (0-2). Patients who received stronger physical restraints underwent rehabilitation training with greater intensity, and a significant difference in training intensity was observed among groups with different physical restraint intensities (χ² = 22.01, df = 10, p = 0.015). For example, patients in the severe restraint group (level 2) received rehabilitation at levels 3-5 more frequently, whereas patients without restraint (level 0) received rehabilitation at lower intensity levels (0-2). Thus, the need for severe restraint may be associated with the intensity of rehabilitation training.

## Discussion

Neurocognitive disorders such as dementia and delirium pose serious challenges to acute care. Establishing an appropriate treatment system is essential for promptly advancing acute treatment and improving the patients’ quality of life. To provide better care for patients with acute neurocognitive disorders, we analyzed two opposing physical activity measures: rehabilitation training intensity and physical restraint intensity. We have previously reported that training intensity among patients receiving dementia care rounds differed significantly by disease group and that patients receiving high-intensity training were more likely to undergo physical restraint [10]. Building on these findings, we conducted a subgroup analysis of patients with cardiovascular disease. Cardiovascular diseases include conditions requiring treatment, such as pacemaker implantation, percutaneous coronary intervention, minimally invasive surgery (endovascular aneurysm repair and endovascular thoracic aortic repair), and laparotomy or thoracotomy. Neurocognitive disorders are serious treatment-impeding factors that may affect the survival prognosis in cardiovascular diseases, including many conditions of high urgency and severity. This subgroup analysis provided important foundational information for acute-phase management.

We predicted that treatments involving greater invasiveness, such as open-chest or abdominal procedures, would result in a higher incidence of neurocognitive disorders than conservative treatment. However, no significant differences in the incidence of neurocognitive disorders were observed between the treatment groups (Table 1). The sample size was small, and the statistical power did not reach 0.8 (data not shown), making the results insufficient for firm interpretation. Interesting findings emerged regarding the number of follow-up visits to the dementia care team. The patients who underwent more invasive treatments generally had more follow-up visits, and a statistically significant difference in the number of follow-up visits was observed between the treatment groups (Figure 2). However, more follow-up visits in the invasive treatment group may reflect longer hospitalization periods rather than greater severity of neurocognitive impairment. This should be considered when interpreting our results. No significant differences were identified in the relationship between rehabilitation training intensity and treatment group or between physical restraint intensity and treatment group. In this study, no statistically significant differences were observed between rehabilitation training intensity or physical restraint intensity and treatment groups. Furthermore, the small effect sizes (Cramer’s V ≈ 0.11) indicate that the magnitude of differences between treatment groups was limited, suggesting that treatment modality itself slightly influenced these aspects of patient management. Moreover, rehabilitation training intensity in this study was assessed using ambulation stages, which reflect the level of mobility training achieved during rehabilitation sessions rather than the overall amount or duration of rehabilitation therapy. Therefore, the lack of differences between the treatment groups should be interpreted within this conceptual framework. However, consistent with our previous report, a significant difference was observed in the relationship between rehabilitation training intensity and physical restraint intensity. Thus, patients who were more active were more likely to receive physical restraints than those who were less active. Logically, patients with greater mobility face a high risk, and physical restraints may be necessary in cases of neurocognitive disorders. This observed association does not imply causality. Reverse causation is possible where more active patients may require restraints for safety reasons, which could also influence the intensity of rehabilitation training. This factor should be considered when interpreting these findings. However, considering that physical restraints are removed during rehabilitation sessions under appropriate supervision, the absence of physical restraints may not necessarily lead to serious incidents. In Japan, physical restraint in hospitals is limited to cases where the following three requirements are met: “imminent danger,” “absence of alternative measures,” and “temporariness” [16]. Alternative interventions aimed at reducing physical restraint include increased staffing levels, staff education, improved documentation systems, covering interventional access sites, and the use of less restrictive devices [17-20]. A more stable one-to-one environment was achieved during rehabilitation, suggesting the usefulness of enhanced staffing in alternative interventions. The prevalence of neurocognitive disorders in acute care hospitals is significant. To the best of our knowledge, this study is the first to report on the characteristics of rehabilitation or physical restraint in patients with cardiovascular disease.

This study has some limitations. First, this was a single-center study. Second, this was a cross-sectional study, making it impossible to determine causality. Third, to review all cases from the perspective of physical activity, the disease classification used was the Ministry of Health, Labor, and Welfare’s classification for rehabilitation. Fourth, the sample size was small. For items with an expected frequency of less than five, the chi-square test may have some issues. However, primary comparisons met the requirements of statistical precision. Finally, specific indications for physical restraint (e.g., fall prevention vs. device removal) were not distinguished; therefore, restraint intensity should be interpreted with caution. Additionally, more active patients may require restraint for safety reasons, which could influence the observed association between rehabilitation training intensity and physical restraint intensity. Furthermore, potential confounding factors, such as patient comorbidities and baseline cognitive status, which may have influenced rehabilitation training intensity and physical restraint intensity, were not fully accounted for.

## Conclusions

This analysis revealed no significant relationship between the onset of neurocognitive disorders and the treatment group. However, the patients who underwent more invasive treatments generally had more follow-up visits, and statistically significant differences in the number of follow-up visits were observed between the treatment groups. No significant differences in rehabilitation training intensity or physical restraint intensity were observed between the treatment groups; however, significant differences were observed in training intensity and physical restraint intensity. In patients with cardiovascular diseases, highly invasive treatments may prolong neurocognitive disorders. Patients receiving stronger physical restraints may tolerate more intensive training, and strengthening staffing levels may reduce the need for physical restraints.
